# Experiment Development and Verification for the Demonstration of Advanced Radiation Shielding in Future Satellite Missions

**DOI:** 10.3390/s26051404

**Published:** 2026-02-24

**Authors:** Nico Gerster, Tobias Dickhut

**Affiliations:** Institute of Aeronautical Engineering, University of the Bundeswehr Munich, 85579 Neubiberg, Germany

**Keywords:** space radiation shielding, dosimetry, fibre-reinforced composites, floating gate MOSFET sensors, radiation environment simulation

## Abstract

This paper outlines the design of the space radiation detection experiment RADS to demonstrate new shielding materials in space during the Athene-1 mission, as well as the Gena-OT1 CubeSat precursor mission. The experiment compares new materials in the form of functional layers integrated into fibre-reinforced composite structures against traditional aluminium shielding. Trapped-particle motion is considered to maximise the exposure of the experiment in space. The radiation sensing units are based on off-the-shelf electronic components. Dosimeters based on a floating-gate MOSFET architecture are used to represent the damage mechanism in electronic devices exposed to space radiation. To account for particle- and energy-specific dose enhancement effects in the silicon of the dosimeters, the concept of a Cobalt-60 equivalent dose is introduced to serve as a calibration baseline. The structural design and software aspects are considered to increase ease of use for future satellite missions. Full 3D radiation simulations were conducted using FastRAD to validate the measurement concept of the sensor units in conjunction with the housing unit and the new shielding material. The experimental design has been verified, showcasing a unique method for evaluating new shielding materials in space.

## 1. Introduction

Space radiation remains a significant design challenge in modern satellite development [1,2,3]. As the demand for lower-cost missions increases alongside expectations for reliability and lifetime, new shielding strategies and materials must be evaluated under realistic conditions. Composite structures with integrated functional layers have emerged as promising candidates for reducing mass while enhancing radiation protection; however, their shielding behaviour remains insufficiently characterised at high energies relevant to Low Earth Orbit (LEO).

The Athene-1 mission, launched within the SeRANIS research programme, serves as an in-orbit technology demonstrator for advanced satellite subsystems, including next-generation communication technologies and multifunctional structural concepts [4,5]. Among these is the radiation shielding experiment RADS, designed to characterise representative composite materials equipped with embedded functional layers. Although multilayer shielding concepts have been extensively investigated in ground-based studies [6,7,8,9], no dedicated experiment has yet flown to evaluate multilayer composite shielding in the space environment, where trapped protons and electrons produce a highly anisotropic and energy-dependent flux that directly constrains experiment geometry, shielding thicknesses, and orientation.

The only closely related work is the design study presented in [10], which introduces a similar radiation-detection concept but focuses on non-structural shielding materials and employs different sensor technologies. In contrast, RADS is specifically targeted at load-bearing composite structures with integrated shielding functionality, enabling the first in-orbit assessment of multilayer structural materials developed for both mechanical and radiation-protection performance.

Ground-based radiation testing provides essential insight but remains fundamentally limited in its ability to reproduce the full complexity of the LEO environment [11]. Facilities capable of delivering particle spectra that faithfully match the broad energy ranges of trapped protons and low-energy electrons are scarce, and conventional accelerator beams, typically optimised for medical or materials-science applications, capture only a narrow subset of relevant conditions. Moreover, practical constraints prevent systematic testing over the wide range of incidence angles encountered in orbit, as expanding the test envelope would be prohibitively demanding. Crucially, ground tests also cannot replicate long-term in-orbit ageing phenomena, including dose-rate effects and time-dependent material interactions that may influence shielding performance. For these reasons, in-orbit measurements remain the only comprehensive means of assessing how advanced shielding materials perform under the full spectrum of space radiation and operational conditions.

To address this challenge, the RADS experiment introduces a controlled, side-by-side comparison of shielding materials under identical geometric and environmental conditions. The experiment consists of a thick-walled enclosure featuring two dedicated measurement windows: one containing a reference aluminium plate and the other a multilayer composite sample. The large wall thickness ensures that the majority of the radiation recorded by the onboard dosimeters enters through these windows, enabling a direct comparison of the attenuation properties of aluminium and the novel material. This approach provides a meaningful evaluation of the advantages that multilayer composites may offer over conventional aluminium structures.

The RADS experiment introduces several novel elements to in-orbit radiation characterisation. First, it applies a Co-60-equivalent dose framework to Floating Gate Dosimeter (FGD-03) measurements, enabling direct interpretation of mixed-field LEO radiation in terms of metal–oxide–semiconductor field-effect transistor (MOSFET)-relevant degradation. Because in-orbit dose-enhancement effects cannot be separated by particle type, expressing damage as an equivalent Co-60 dose provides a practical and qualification-aligned baseline for comparing shielding performance and estimating component lifetimes.

Second, RADS implements a novel measurement concept specifically designed for the validation of shielding materials. The paper outlines the complete design philosophy, including the geometric layout and the selection of shielding material thicknesses across the experiment based on ShieldDose-2 predictions. Together with a fixed flight-direction orientation that exploits LEO proton anisotropies, this configuration provides well-isolated, material-specific dose measurements, as confirmed through detailed 3D radiation-transport simulations.

Third, RADS provides the first on-orbit evaluation of multilayer shielding materials based on carbon fibre reinforced polymer (CFRP) laminates incorporating bespoke functional layers. This creates a unique opportunity to assess these lightweight, application-driven materials under the full LEO spectrum, offering insight into their long-term shielding behaviour and informing future design iterations.

## 2. Materials and Method

### 2.1. Mission Environment and Constraints

The Athene-1 mission will operate in a sun-synchronous orbit (SSO) at 500 km to 600 km altitude, where the radiation environment is dominated by particles trapped in Earth’s magnetic field. To quantify the expected conditions, the AE9 (electrons) and AP9 (protons) models were used, based on a 95th percentile confidence level to represent conservative environmental conditions. Lower percentile spectra were found to affect the absolute total ionising dose but did not significantly influence the qualitative conclusions regarding payload performance or shielding design. Very low percentile spectra were not considered, as they do not represent nominal or worst-case mission-relevant conditions. These models predict trapped protons with energies up to ∼400 MeV and trapped electrons up to ∼7 MeV in the targeted orbit. Galactic Cosmic Rays (GCRs) contribute only a minor fraction of the accumulated ionising dose at this altitude because of geomagnetic shielding [12] and short exposure times in polar regions where geomagnetic shielding is limited. The AE9/AP9 spectral fluxes generated for this orbit serve as input for the later ShieldDose-2 dose–depth analysis used to dimension the experiment housing.

The motion of the trapped particles has direct consequences for experiment geometry and orientation. Charged particles follow a combination of gyro-motion around magnetic field lines, bounce motion between mirror points near the poles, and longitudinal drift [13]. A satellite in SSO similarly progresses along repeated south–north and north–south passes, resulting in an advantageous alignment: the flight-direction facing surface of the satellite is continuously exposed to particles arriving with pitch angles that intersect the orbit plane. This enhances the incident proton and electron flux on that surface. Additionally, the orbit frequently transits the South Atlantic Anomaly (SAA) [14], where geomagnetic shielding is weakest and proton fluxes peak by several orders of magnitude. During these passages, the flight-direction surface receives disproportionately high trapped-proton exposure, improving the statistical significance of the measurements.

The geometric design of the RADS experiment is further constrained. These design constraints heavily influence the design of the experiment. These constraints include:

1. The experiment must be platform-agnostic, with a maximum 2U form factor for precursor CubeSat flights, while remaining compatible with the larger Athene-1 bus [15].
2. The experiment must incorporate a reference dose measurement through a piece of aluminium with the same areal density as the novel shielding material, as well as the same ’window size’ such that the same fraction of solid angle receives radiation. This serves to compensate for platform, space weather, and orientation-based influences on dosimetric measurements. The vicinity of the two measurement conditions allows for extraneous influences to be controlled.
3. The design must maximise the fraction of radiation penetrating the material sample, to ensure higher dose measurements occur, despite better shielding performance of the material. This is required to more rapidly detect statistically significant differences in the shielding performance of the novel shielding material compared with the reference aluminium.
4. The mass of the experiment must be minimised, yet the housing requires sufficiently thick walls to ensure that the dominant fraction of radiation enters the detectors only through the designated measurement windows, thereby preserving scientific interpretability.
5. The experiment must be able to perform in the space environment, meaning it must withstand extreme temperature differences and other damage mechanisms such as atomic oxygen.

### 2.2. Dosimetry Method and Sensor Calibration

The RADS experiment employs Floating-Gate Dosimeter (FGD) sensors of the type FGD–03F as the primary radiation-sensing elements. Floating-gate MOSFET dosimeters have been extensively characterised in mixed proton, ion, and gamma environments [16,17,18], and similar technologies have flown on deep-space missions such as the lunar flyby in [19]. Their operating principle makes them particularly suitable for engineering applications, as the damage mechanisms they experience are representative of those occurring in MOSFET-based electronic components used throughout modern satellite systems.

Each FGD–03F dosimeter provides a frequency signal which decreases as the sensor is exposed to ionising radiation. During operation, the experiment records the raw frequency, which provides a highly stable and low-power proxy measurement for total ionising dose (TID). The conversion from frequency shift to dose is performed during ground processing, providing maximal flexibility when analysing the in-orbit data.

A pre-flight calibration was performed on all FGD–03F sensors integrated into the RADS experiment. The calibration was carried out at the Fraunhofer Institute for Technological Trend Analysis (Fraunhofer INT) using a Cobalt-60 irradiation facility. Co–60 is the standard reference radiation source for TID testing in electronics, as formalised in ESCC-22900 [20] and MIL-STD-883 Test Method 1019 [21].

To maximise the usable in-flight dose range of the dosimeters, the calibration was intentionally conducted at a low total dose of approximately 30 rad. This ensures that the sensors remain within their linear response regime throughout the expected lifetime of the Athene-1 mission. During calibration, the true dose was monitored using an ionisation chamber positioned adjacent to the dosimeters, allowing the sensor frequency shift to be directly correlated with the absorbed dose. A linear regression between measured dose and observed frequency shift was generated for each device.

The FGD–03F sensors are continuously read out by the RSU microcontroller, and each measurement frame includes the sensor frequency and a local temperature measurement. All data are stored and forwarded without onboard conversion, allowing calibration curves, correction factors and statistical methods to be applied during ground analysis.

The FGD response exhibits a known temperature dependence, which is corrected on the ground using a lookup table (LUT) derived from pre-flight characterisation tests. The high accuracy and resolution of the dosimeters ensure that even early in the mission, differences in test conditions can be detected, and as measurements accumulate over time, these differences will grow and become increasingly statistically significant.

While the calibration is performed with Co–60 gamma radiation, the in-orbit radiation field consists primarily of trapped protons and electrons. FGD sensors, like other MOSFET-based devices, exhibit radiation-response variations depending on particle species, energy, and interaction mechanism. These differences produce “dose-enhancement” effects when comparing proton-induced damage to Co–60 gamma irradiation [22,23].

Holmes-Siedle and Adams identified several effects in the silicon oxide of MOSFET-based dosimeters, including the photoelectric effect, electron scattering, heavy-particle stopping, electron–hole recombination, and field effects [22]. The contribution of these effects depends on the particle type or the photon forming the source of radiation. Further studies also observed dose-enhancement effects in Co-60 calibrations, particularly for high-energy protons [18].

Because these enhancement factors cannot be resolved in flight, the RADS experiment reports an equivalent Co–60 dose. This represents the dose from a Co–60 source that would produce the same threshold-shift damage as observed under the complex low-Earth-orbit particle environment and serves as an effective degradation metric rather than a physically equivalent absorbed dose. Reporting in Co–60 equivalent dose is consistent with the definition of TID limits for space electronics and enables direct comparison between measured on-orbit degradation and qualification standards for MOSFET-based components.

This approach provides valuable insight into the shielding effectiveness of the tested materials and the expected degradation of satellite electronics exposed to similar radiation environments.

### 2.3. 3D Radiation Transport Simulations

To evaluate the measurement concept of the RADS experiment and to quantify the contribution of primary and secondary radiation entering through the measurement windows, detailed 3D radiation transport simulations were performed using the TRAD software suite [24]. The simulations were carried out using the reverse Monte Carlo mode, which allows efficient estimation of dose deposition at small detector volumes by tracing particle histories backwards from point-like detectors.

The full CAD model of the RADS experiment was imported into TRAD without geometric simplification. All structural features, including the exterior aluminium cage, internal web separating the two RSUs, and the sensor board, were preserved.

For the purpose of producing conservative (worst–case) estimates and to reduce model complexity, both the reference window and the material sample window were modelled as 2 mm aluminium plates. In the flight hardware, the aluminium reference window thickness is approximately 1 mm, and would admit a higher particle and dose transmission. Thus, the simulation setup underestimates the window ’transparency’ and therefore yields conservative results for the isolation of the measurement regions. The PCBs of each RSU were modelled as standard FR4, and the dosimeter chips were explicitly included in the CAD model. Reverse Monte Carlo point detectors were placed at the geometric centres of the silicon die volumes in each sensor.

The incident particle environment was defined using the same proton and electron spectra applied in the ShieldDose 2 analyses. Differential flux spectra predicted for the 500 km to 600 km Sun–synchronous orbit were taken from the AP9 and AE9 models [25]. Protons with energies up to several hundred MeV and electrons up to 7 MeV were included.

In the reverse Monte Carlo configuration, TRAD assumes an isotropic 4π steradian distribution of the incoming particles surrounding the experiment. This approach neglects the directional anisotropy associated with charged particle motion along geomagnetic field lines. As a result, the simulation produces a conservative, worst–case estimate by including radiation contributions that would not be encountered for all spacecraft orientations in flight.

Monte Carlo simulations were performed with particle histories in the order of several million per particle species and scenario, resulting in relative statistical uncertainties below 5% at the detector locations. Error bars are omitted from the figures for clarity. This uncertainty level was considered sufficient to ensure the robustness and interpretability of the presented results.

A sensitivity analysis with respect to shielding thickness was not performed, as nominal manufacturing tolerances are well below the thickness variations required to produce a meaningful change in dose for the considered configuration. Consequently, thickness uncertainty is not expected to materially affect the conclusions of this study.

The primary objective of the simulations was to demonstrate that, with the selected wall and window thicknesses, the majority of the dose recorded by each dosimeter arises from radiation passing through the corresponding measurement window. In particular, the simulations were used to verify:

- that the thick aluminium sidewalls suppress lateral penetration and ensure that most contributing particles arrive through the windows;
- that “cross-contamination” between the reference and material–sample RSUs is minimal due to the shielding effect of the central aluminium web and geometric separation;
- that the ratio between window contributions is preserved even under conservative worst–case assumptions.

A secondary objective was to obtain a first–order estimate of the dose received by components on the internal PCB, including the microcontroller on each RSU. This provides valuable preliminary information regarding total ionising dose exposures in orbit. Simulation outputs included:

- the cumulative absorbed dose at each point detector;
- the spatial distribution of contributing particle trajectories;
- visualisation of particle paths to illustrate dominant entry routes;
- relative dose contributions from the reference window, sample window, and lateral directions.

All Monte Carlo indicators tracked by TRAD were monitored until convergence. Histories were accumulated until the fractional statistical uncertainties for all scored quantities reached acceptable limits. Although no external analytical validation was performed, the internal convergence metrics ensured numerical stability of the results.

## 3. Results

### 3.1. RADS Experiment Design

The RADS experiment was developed to characterise novel multilayer shielding materials in low Earth orbit under well-defined and scientifically meaningful conditions. Its design requirements were derived from the mission environment, platform constraints, and the need to ensure that the measured dose originates predominantly from radiation passing through the dedicated material windows.

The performance of the functionalised radiation shielding composites was estimated using MULASSIS. These simulations revealed that these advanced shielding materials offer a 30% reduction in TID compared to aluminium. This increased shielding is detectable at high resolution in the sensors early on but becomes increasingly statistically significant over time. With an intended mission lifetime exceeding 1 year, a significant difference in the dose measurements can be anticipated.

The baseline design, therefore, employs a thick-walled enclosure with two dedicated windows: one exposing a reference aluminium sheet and the other exposing a multilayer shielding sample. The remaining surfaces are sufficiently thick to minimise the lateral penetration of high-energy particles.

Together, these mission-specific constraints define the geometric boundary conditions for the later dose–depth simulations and directly inform the mechanical layout of the experiment described in the following sections.

Each radiation sensor is a 5 × 5 mm solid-state dosimeter, for which the angular acceptance strongly influences the accumulated statistics. To maximise the range of incidence angles sampled in flight—a regime that cannot be efficiently reproduced in ground testing—the measurement windows are intentionally made large relative to the sensor area. This allows particles entering over a broad solid angle to reach the sensitive volume without being intercepted by the housing walls. A second measurement window placed on the zenith-facing side ensures that both shielding configurations also receive comparable GCR exposure, while the nadir-facing surface is reserved for mechanical and electrical interfaces because it is naturally attenuated by Earth.

The experiment consists of a machined 6061-T6 aluminium housing, based on its extensive track record [26], enclosing two radiation sensing units (RSUs). The housing incorporates two apertures in the lid: a reference window consisting of a thinned aluminium section and a measurement window accommodating the composite shielding sample. The sample is mechanically retained by rivets to ensure dimensional stability throughout launch and orbital operations.

**Figure 1:**
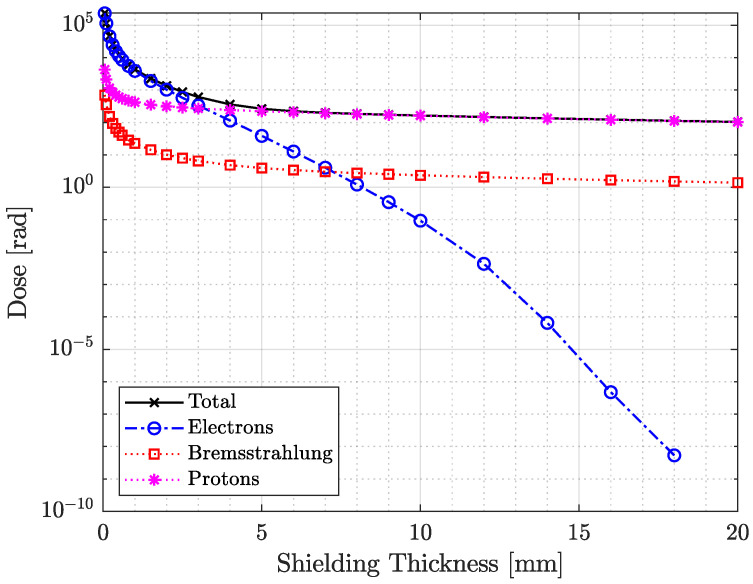
Total dose for Athene-1 mission predictions for varying shielding thickness according to ShieldDose-2. The total dose, as well as the components, are shown. The total dose curve informed the selection of the RADS experiment cage wall thickness.

A minimum wall thickness was derived from ShieldDose-2 dose–depth curves generated using the AE9/AP9 spectral flux predictions (see Figure 1). A thickness of 4 mm was selected as a compromise between suppressing lateral penetration of high-energy trapped particles and maintaining low mass and compact dimensions relevant for the CubeSat format. This ensures that the majority of radiation reaching the detectors passes through the intended windows rather than the surrounding structure, preserving the interpretability of the shielding comparison.

Although the payload mass budget of the Athene-1 spacecraft is stringent, the design remains compliant while retaining the structural integrity required to withstand launch loads and the on-orbit environment. The geometry also adheres to a maximum 2U envelope, enabling compatibility with CubeSat precursor missions and simplifying accommodation on a wide range of platforms without redesign.

An internal structural web separates the two RSUs, providing mechanical isolation and dosimetric decoupling so that the measurements behind the aluminium reference and the composite sample are not mutually influenced. All mechanical components incorporate venting holes sized according to ESCC guidelines [27] to avoid entrapped gas during ascent; their placement prevents directed flow toward solar panels or sensitive optics. To support repeated integration and testing cycles, all threaded holes employ self-locking helicoil inserts, and the lid/base interface features an asymmetrical interlocking profile that prevents incorrect assembly and guarantees correct window–sensor alignment.

**Figure 2:**
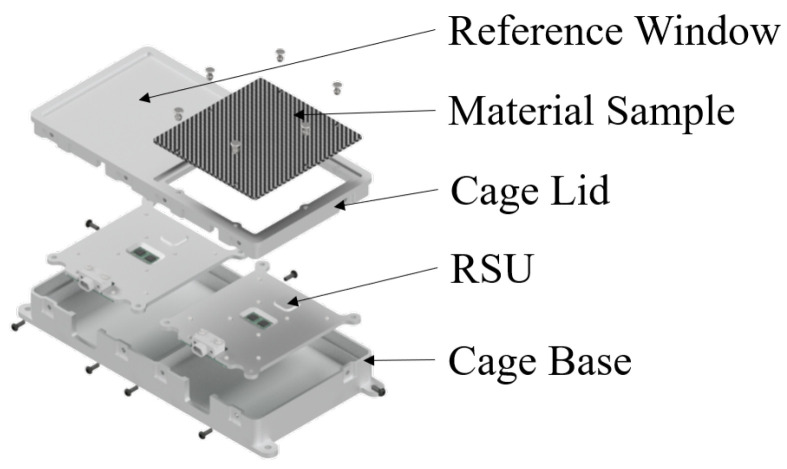
Exploded view of the RADS experiment for the Athene-1 mission showing the cage, radiation sensing units, and material sample.

An exploded view of the RADS experiment is shown in Figure 2, showing all components and their arrangement. This reveals the overall functioning principle of the RADS experiment.

The RSUs are designed for continuous and autonomous operation without requiring any dedicated software or supervisory functions from the spacecraft. The RSUs have built-in power conditioning to allow for an unregulated power supply to the experiment to be used. Power consumption is kept as low as possible to ensure that the experiment can operate throughout the mission without duty cycling.

Each RSU is a self-contained system that includes a radiation sensor, a microcontroller, power conditioning, and a communication interface. The units are electrically independent from one another to prevent fault propagation. Temperature-critical components were selected to operate reliably across –20 °C to +80 °C, ensuring survivability in the expected orbital thermal environment under passive thermal control.

The microcontroller is an ARM Cortex-M4 device previously used in multiple LEO missions and subjected to total-ionising-dose testing [28]. It is complemented by an external watchdog timer to mitigate latchup-induced functional interruptions and by an external radiation-tolerant flash memory that enables reflashing firmware in case of anomalies. Additional protection circuitry guards against overvoltage and overcurrent events.

To capture statistically significant radiation dose data under varying orbital conditions, the measurement cadence is programmable and can range from 1 Hz to 1 sample per minute, allowing optimisation for mission duration, expected flux, and power constraints. All measurements are processed by the microcontroller and stored in a JSON file, which can be easily parsed on the ground. The microcontroller then forwards the packaged data to the experiment data handling unit of the satellite, where it is stored and downlinked at the appropriate scheduled intervals.

Communication is implemented using the OpenCyphal protocol over a UART/RS-485 physical interface, providing robust telemetry transfer suited for space applications. The entire experiment is designed as a plug-and-operate subsystem requiring no platform-specific software beyond standard communication handling.

**Figure 3:**
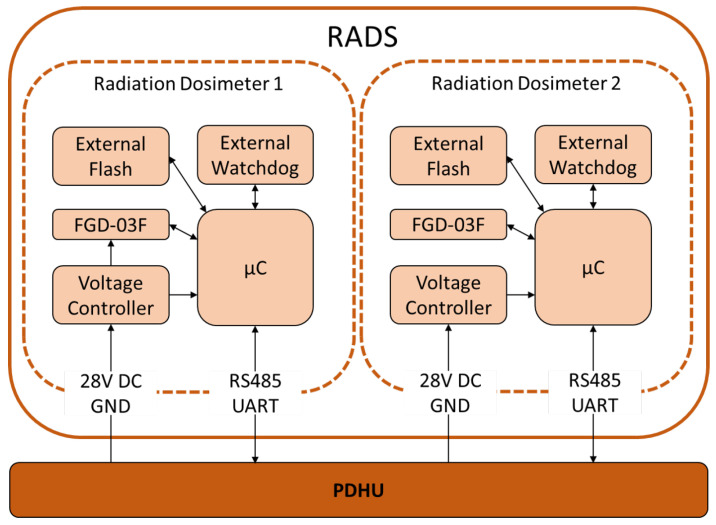
Schematic diagram of the RADS experiment, showing both sensor units with power and data interfaces. Furthermore, the data flow and communications within each sensor unit are shown.

Shown in Figure 3 is a block diagram of the RADS experiment. This shows the way in which each RSU is constructed and interfaces with the experiment data handling unit of the satellite bus.

### 3.2. 3D Radiation Transport Simulation Results

Reverse and forward Monte Carlo radiation transport simulations were performed to evaluate the measurement concept of the RADS experiment and to verify that the majority of the dose is incident through the two dedicated measurement windows. All simulations were carried out using the TRAD toolchain [24].

To provide external verification of the radiation transport analysis, supplementary simulations were performed using the MULASSIS framework with simplified shielding models. In MULASSIS, aluminium layers of varying thickness were modelled to represent key structural elements of the payload, including the detector measurement window, cage walls, rear enclosure, and additional assumed spacecraft shielding. As MULASSIS assumes laterally infinite planar layers, the comparison was performed by matching equivalent material thicknesses rather than detailed geometric components.

The radiation environments were derived from the AP9/AE9 trapped particle models, consistent with those used in the three-dimensional TRAD simulations. The primary comparison metric was absorbed dose. The resulting MULASSIS and Shieldose-2 dose estimates were found to be in agreement with the TRAD results, confirming that the simulation setup and material definitions were physically consistent.

TRAD was retained as the primary simulation tool for payload design, as it enables the analysis of complex three-dimensional geometries and detailed component-level shielding effects. MULASSIS and Shieldose-2 were used solely for verification purposes to ensure that the TRAD simulations were correctly configured and that the predicted dose levels were meaningful.

**Figure 4:**
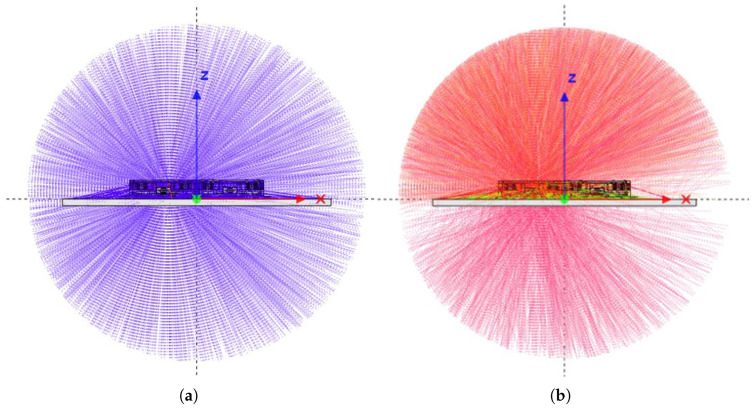
Reverse Monte Carlo particle tracks for protons and electrons in the RADS experiment. Proton tracks show no significant directional dependence in track density. In contrast, electron tracks exhibit a markedly higher density incident from the top over a wide range of angles, as well as from the rear, indicating that electron radiation is strongly attenuated by the RADS housing geometry. (**a**) Proton tracks. (**b**) Electron tracks.

Figure 4 shows example track visualisations for protons and electrons. For electrons, the simulations confirm a strong dominance of particles entering through the measurement windows; this is expected due to the relatively low penetration depth of electrons compared to the wall thickness of the experiment. Only a small fraction of the electron dose enters through the sides or back of the housing. The back face admits slightly more electrons than the lateral faces, but this contribution will be further attenuated by the satellite structure on which RADS is mounted.

Protons, owing to their substantially higher energies (up to several hundred MeV in the mission orbit), exhibit a more uniform angular distribution within the experiment. However, their total dose contribution is much lower than that of trapped electrons, meaning the measurement concept remains dominated by window-entry dose. Overall, the simulations confirm minimal cross-contamination between the reference and material-sample measurement volumes.

High-energy protons, while contributing a smaller fraction of the total ionising dose, are known to be a major driver of long-term reliability degradation through displacement damage and single-event effects. The present study, therefore, focuses on total ionising dose, as this is the damage mechanism most amenable to mitigation by passive shielding. High-energy particle-induced effects are comparatively difficult to reduce through shielding under the strict mass constraints inherent to space systems.

As the payload is mounted facing the flight direction, it will maximally exploit the anisotropic flux of charged particles in orbit. The selected orbit will approximately follow the magnetic field lines, with north- and south-facing passes. As such, the isotropic 4π particle flux assumed in the simulations represents a worst-case condition if the satellite were to lose attitude control and tumble. With attitude control, radiation will enter via the detection windows due to the geometric design of the payload, and secondly, due to the exploitation of anisotropic flux in orbit, coupled with orbit selection and payload accommodation.

### 3.3. Directional Dose Contribution (Forward Monte Carlo)

**Figure 5:**
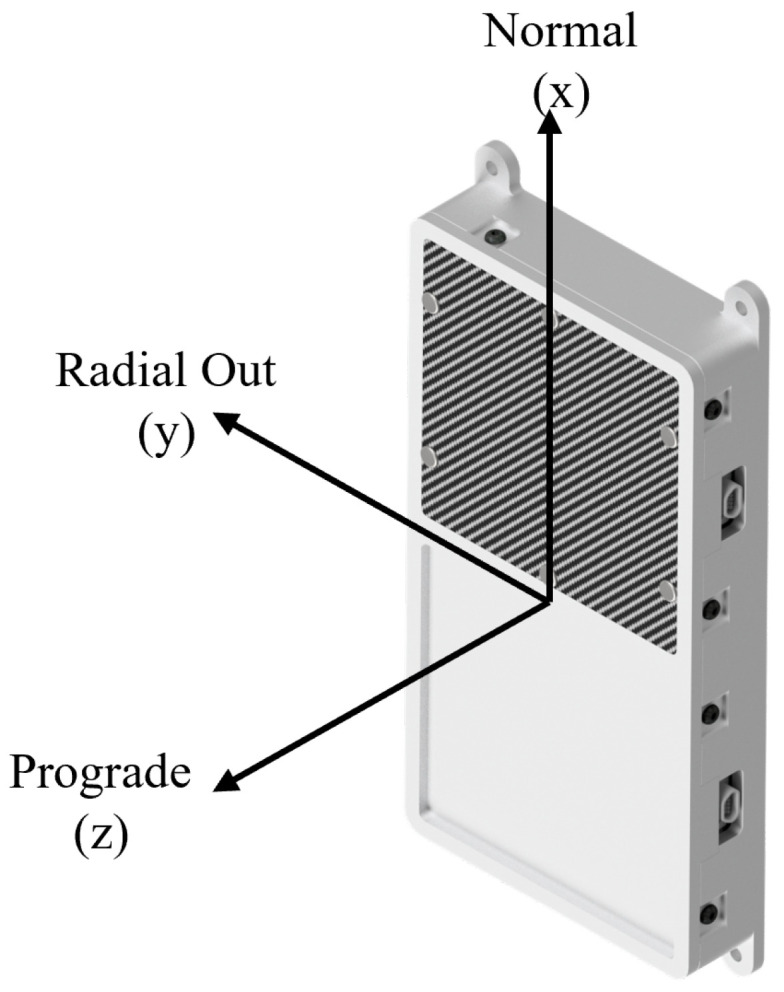
Definition of forward-simulation source planes. Anti-normal and radial-in faces are omitted for clarity due to symmetry and negligible expected radiation from Earth-facing directions.

To corroborate the reverse Monte Carlo analysis, forward simulations were performed using four planar source surfaces surrounding the experiment. These surfaces represent the dominant radiation incidence directions in the sun-synchronous orbit: the prograde face (flight direction), the retrograde face, the radial-out face, and the normal face. The coordinate system and naming convention are shown in Figure 5. Anti-normal and radial-in directions were omitted, as the experiment geometry is symmetric and radiation originating from Earth would unrealistically overestimate dose contributions from below.

Each source plane was simulated independently using the AP-9/AE-9 differential spectra and random incidence angles. Table 1 summarises the results for electron-induced total dose. As each simulation assumes the full mission flux originates from a single plane, the absolute dose values appear elevated; however, the relative contributions clearly show that more than 90% of the electron dose originates from the prograde direction, where the measurement windows are located. This result confirms the geometric validity of the measurement concept.

### 3.4. Dose to Critical Electronic Components

**Figure 6:**
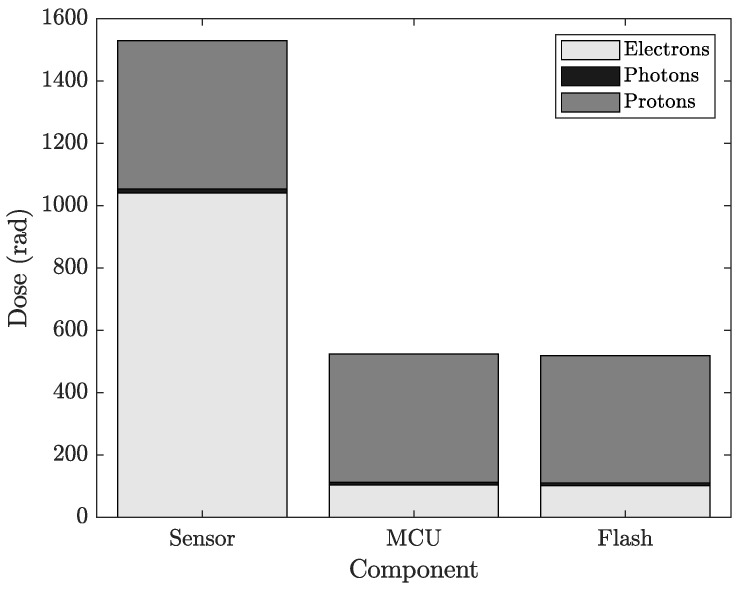
Simulated accumulated dose for key electronic components on the RSU. The proton-induced dose is nearly identical for all ICs due to the high penetration of protons, whereas the electron-induced dose is strongly suppressed by the PCB and local shielding.

Reverse Monte Carlo simulations were also used to estimate the accumulated dose in critical integrated circuits (ICs) located on each RSU. The approximate IC positions were extracted from the CAD geometry and used as point detectors. Figure 6 summarises the predicted mission-integrated dose for the microcontroller, the external flash memory, and the FG dosimeter itself.

The simulations show that the microcontroller and external flash receive a substantially lower dose than the dosimeters, confirming that the local shielding strategy on the RSU is effective. Proton-induced dose is similar across all devices, reflecting the difficulty of stopping high-energy trapped protons. This highlights the importance of robust latch-up protection, even though the overall proton dose remains far below the electron-induced contributions.

Overall, the simulation campaign validates the core measurement principle of RADS: the dose measured by each dosimeter is dominated by radiation passing through its respective measurement window, with minimal unwanted dose from other directions, and component-level doses remain within safe operational margins.

The results presented demonstrate that the RADS experiment design effectively achieves its primary objective: to characterise the shielding performance of novel materials in low Earth orbit. The combination of a thick-walled housing with dedicated measurement windows ensures that the majority of the radiation measured by each dosimeter passes through the intended material sample rather than through the surrounding structure. Both reverse and forward Monte Carlo simulations confirmed the robustness of this measurement principle and highlighted the minimal cross-contamination between the reference and test materials.

The use of FG dosimeters, combined with the concept of an equivalent Co-60 dose, provides a practical and meaningful way to quantify radiation effects in the complex LEO environment. Given the mixed particle spectrum, the energy range, and differences in interaction mechanisms between electrons and protons, this approach allows the measured dose to be directly related to standard total ionising dose metrics used in electronics design. This ensures that the measurements are not only scientifically meaningful but also directly applicable to the assessment of radiation shielding for electronic devices in space.

Overall, the RADS experiment represents a compact, low-mass, and autonomous solution for in-orbit characterisation of shielding materials. Its design is flexible enough to accommodate a variety of material samples and can be integrated with small satellite platforms, enabling comparative studies under realistic space radiation conditions.

### 3.5. Future Work

The next step for the RADS concept is the deployment of the experiment on the Gena-OT1 and Athene-1 mission and subsequent in-orbit demonstration of shielding materials. This will allow direct validation of the predicted dose attenuation through the measurement windows and provide the first in-space comparison between conventional aluminium shielding and novel composite or functional materials.

The resulting data will inform both the selection and optimisation of shielding strategies for future satellite missions and enable further refinement of the RADS experiment for subsequent applications.

## 4. Conclusions

The RADS experiment has been designed and validated through detailed Monte Carlo simulations to provide a reliable and flexible platform for the in-orbit characterisation of advanced radiation shielding materials. The design and implementation are specific to the deployment in a sun-synchronous orbit. The simulations confirm that the dominant fraction of incident particles reaches the dosimeters through the intended measurement windows, ensuring that differences in recorded dose arise primarily from the shielding materials and thicknesses under test. Combined with temperature-compensated FGMOS dosimetry and the equivalent Co-60 dose framework, the system quantifies mixed-field LEO radiation in a form directly relevant to electronic component degradation and spacecraft design.

The upcoming flights of the precursor mission, launched on Transporter 15 on the 18 November 2025, and the subsequent Athene-1 mission in late 2026 will deliver the first in-space demonstration of multilayer composite shielding materials embedded within structural CFRP. Time-resolved dose–accumulation curves will be obtained for both aluminium reference samples and the CFRP laminate incorporating bespoke functional layers, enabling direct comparison of shielding efficiency and assessment of long-term material performance in the LEO environment. While minor cross-contamination between neighbouring measurement locations is expected, it remains well characterised and does not impede material-specific interpretation.

The dataset produced by these missions will support the validation and optimisation of novel shielding materials, informing the refinement of functional-layer compositions for both performance and economic viability. More broadly, RADS establishes the first dedicated in-orbit platform for evaluating multilayer composite shielding concepts, contributing to improved spacecraft radiation protection strategies and advancing the development of lightweight, radiation-tolerant structures for future missions.

## Figures and Tables

**Table 1:** Forward Monte Carlo total dose contributions for trapped electrons. Values show the fraction of dose entering each face, assuming the full mission flux arrives exclusively from the respective direction.

Detector Plane Normal Vector	Dose	
	Electrons [rad]	TID [%]
Prograde	$1.85\times 10^{3}$	90.7
Retrograde	$1.54\times 10^{2}$	7.5
Radial-Out	$1.06\times 10^{1}$	0.9
Normal	$1.48\times 10^{1}$	0.9

## Data Availability

The data supporting the findings of this study are available from the corresponding author upon reasonable request.

## Abbreviations

The following abbreviations are used in this manuscript:

LEO	low Earth orbit
SeRANIS	Seamless Radio Access Networks for Internet of Space
RADS	radiation shielding
MOSFET	metal–oxide–semiconductor field-effect transistor
FGD	floating gate dosimeter
GCR	galactic cosmic radiation
SSO	sun synchronous orbit
SAA	south atlantic anomaly
RSU	radiation sensing unit
ESCC	European Space Components Coordination
PCB	printed circuit board
FR4	flame retardant 4
TID	total ionising dose
LUT	look up table
CAD	computer-aided design
